# Anti-Müllerian hormone as a predictor of the number of oocytes obtained during in vitro fertilization treatments

**DOI:** 10.5935/1518-0557.20240049

**Published:** 2024

**Authors:** Ana Braga Reis, Carla Leal, Márcia Barreiro, António Tomé, Emídio Vale-Fernandes

**Affiliations:** 1 Unidade de Saúde Familiar Porta do Sol, Unidade Local de Saúde de Matosinhos, Matosinhos, Portugal; 2 ICBAS - School of Medicine and Biomedical Sciences, UMIB - Unit for Multidisciplinary Research in Biomedicine, University of Porto, Porto, Portugal; 3 Centro de Procriação Medicamente Assistida/Banco Público de Gâmetas, Centro Materno-Infantil do Norte Dr. Albino Aroso (CMIN), Unidade Local de Saúde de Santo António (ULSSA), Porto, Portugal; 4 Departamento da Mulher e da Medicina Reprodutiva, Centro Materno-Infantil do Norte Dr. Albino Aroso (CMIN), Unidade Local de Saúde de Santo António (ULSSA), Porto, Portugal; 5 ITR - Laboratory for Integrative and Translational Research in Population Health, University of Porto, Porto, Portugal

**Keywords:** anti-Müllerian hormone, *in vitro* fertilization, intracytoplasmic sperm microinjection, oocytes retrieved, ovarian response, ovarian reserve

## Abstract

**Objective:**

To evaluate the correlation between serum anti-Müllerian hormone (AMH) and the number of oocytes retrieved after controlled ovarian stimulation for in vitro fertilization treatments and determine cut-off values predictive of poor and high response to stimulation.

**Methods:**

It was performed a retrospective observational study that included 1003 cycles of controlled ovarian stimulation carried between February 2017 and December 2023 at a Medically Assisted Procreation Centre. The exclusion criteria were the following: serum AMH levels obtained more than 6 months prior to the start of the ovarian stimulation, the presence of a single ovary, non-Caucasian ethnicity, a controlled ovarian stimulation cycle performed for the purpose of oocyte donation or fertility preservation, a documented diagnosis of endometriosis, a documented history of ovarian surgery and the absence of essential data for the study in the medical records (absence of the number of oocytes obtained or the AMH value). Poor response to stimulation was defined as ≤ 3 oocytes retrieved, and high response was defined as > 15 oocytes. The correlation between variables was calculated using Spearman’s correlation test and cut-off values were determined using ROC (Receiver Operating Characteristic) curves.

**Results:**

AMH exhibited a significantly positive correlation with the number of oocytes retrieved (Spearman’s correlation coefficient = 0.60, *p*<0.01). The predictive cut-off for poor ovarian response was 0.72 ng/mL (specificity of 95.13%, sensitivity of 43.23%), and the predictive cut-off for high ovarian response was 4.77 ng/mL (specificity of 89.86%, sensitivity of 38.22%).

**Conclusions:**

Serum AMH proved to be a good predictor of the ovarian response to controlled ovarian stimulation for in vitro fertilization treatments, which makes it useful in supporting clinical decision-making. However, it should not be used as an absolute discriminator of poor or high ovarian response.

## INTRODUCTION

The World Health Organization estimates that infertility affects around 10 to 15% of couples worldwide. Although there is no specific data for Portugal, several studies show that in Western countries infertility affects around 14% of the population, leading many couples to resort to Assisted Reproductive Technology (ART) techniques (DGS, 2010).

However, ART techniques do not guarantee success. In the 2015 Portuguese report on ART activity it is shown that 5886 cycles of in vitro fertilization (IVF) / Intracytoplasmic Sperm Microinjection (ICSI) were started, resulting in 1546 clinical pregnancies and 1379 live newborns (CNPMA, 2015).

The ovarian response to controlled ovarian stimulation is one of the crucial points for the success of second-line ART techniques. Predicting ovarian response prior to the start of stimulation is of great importance, as it not only allows the stimulation protocol to be adapted to maximise the predicted response, but also allows couples to be better advised.

A poor response to controlled ovarian stimulation usually results in a reduced number of oocytes. However, the classification of patients as “poor responders” is not consensual (Ferraretti & Gianaroli, 2014). To address the lack of a standardised definition, the European Society of Human Reproduction and Embryology (ESHRE) published the Bologna criteria, which aimed to define which women should be considered poor responders (Ferraretti *et al.*, 2011), and the POSEIDON (Patient Oriented Strategies Encompassing IndividualizeD Oocyte Number) group also proposed a new classification (Esteves *et al.*, 2019).

Both classifications place great importance on the quantitative assessment of a woman’s ovarian reserve. Various markers of ovarian reserve have been studied, in particular the antral follicle count (AFC) by transvaginal ultrasound and serum anti-Müllerian Hormone (AMH) (Ferraretti *et al.*, 2011; Nelson, 2013; Li *et al.*, 2016; Liu *et al.*, 2023). When compared to each other, AMH and AFC have very similar predictive potential, however, the substantial intraand inter-observer variability and reduced reproducibility of AFC have favoured the use of AMH (Satwik *et al.*, 2012; Vural *et al.*, 2014).

An exaggerated ovarian response (generally defined as obtaining a high number of oocytes) (Arce *et al.*, 2014) can also lead to a poor prognosis (Vale-Fernandes *et al.*, 2023a). The fertilization rate is lower due to a higher proportion of immature oocytes, and the live birth rate is also negatively affected due to the deleterious effect of high serum oestradiol levels on embryo implantation after transfer (Kok *et al.*, 2006; Sunkara *et al.*, 2011). An exaggerated ovarian response can also lead to the development of Ovarian Hyperstimulation Syndrome (OHSS) (Lekamge *et al.*, 2007), a potentially severe condition.

One of the criticisms pointed to the mentioned ovarian reserve markers is the absence of cut-off values predictive of poor or exaggerated ovarian response (Esteves *et al.*, 2018). Regarding AMH specifically, some of the reasons are inter-laboratory and inter-individual variability (Nelson & La Marca, 2011).

Individualising the controlled ovarian stimulation protocol according to the predicted ovarian response seems to be the best way to maximise the prognosis of the ART techniques and reduce the associated iatrogenic risks (Fauser *et al.*, 2008; Silva *et al.*, 2016). Therefore, the study of ovarian reserve markers and the determination of precise cut-off values, adapted to the population of each reproductive medical centre, is of great importance.

The aim of this study is to assess the correlation between serum AMH values and the number of oocytes obtained after controlled ovarian stimulation for IVF treatments, and determine the predictive cut-off values for poor and exaggerated response to stimulation, adapted to the population of the study. A comparison will also be made between the cut-offs determined and those presented in the Bologna criteria and in the POSEIDON criteria.

## MATERIAL AND METHODS

### Study design

A retrospective observational cohort study was carried to assess the correlation between serum AMH values and the number of oocytes obtained after controlled ovarian stimulation for IVF treatments, and to determine which AMH cut-off values are predictive of poor and exaggerated ovarian response. The study was approved by the Institutional Ethics Committee. The principles of the Declaration of Helsinki were followed.

The relevant medical data was obtained from the hospital’s records. The dosing of AMH values was carried using the Beckman Coulter AMH Gen II kit, which employs an enzyme-linked immunosorbent assay (ELISA) technique, particularly a two-site sandwich ELISA (Nelson & La Marca, 2011). All AMH values in this article were presented in ng/mL.

### Participants

After applying the inclusion and exclusion criteria, a total of 1003 controlled ovarian stimulation cycles were included in the analysis.

The inclusion criteria were as follows: IVF/ICSI cycles performed between February 2017 and December 2023, at the Unidade Local de Saúde de Santo António (ULSSA) Medically Assisted Procreation Centre.

The following exclusion criteria were defined: serum AMH levels obtained more than 6 months prior to the start of the ovarian stimulation, the presence of a single ovary, non-Caucasian ethnicity, a controlled ovarian stimulation cycle performed for the purpose of oocyte donation or fertility preservation, a documented diagnosis of endometriosis, a documented history of ovarian surgery and the absence of essential data for the study in the medical records (absence of the number of oocytes obtained or the AMH value).

The stimulation protocol and the dose of gonadotrophins used in controlled ovarian stimulation cycles were chosen based on the experience of the reproductive medicine specialists, taking into account clinical (age, previous treatments), analytical (AMH) and ultrasonographic (AFC) aspects.

### Aims of the study

The first objective was to assess the correlation between serum AMH values and the number of oocytes obtained after controlled ovarian stimulation.

The second objective was to determine AMH cut-off values for poor response to stimulation and exaggerated response. Poor response to stimulation was defined as ≤ 3 oocytes obtained (Ferraretti *et al.*, 2011) and exaggerated response was defined as >15 oocytes obtained (Broekmans, 2019).

### Statistical analysis

All statistical analyses were carried out using IBM SPSS Statistics software (version 26). Descriptive statistics were used to define the demographic data of the population (woman’s age, body mass index (BMI), serum AMH value, ART technique - IVF/ICSI, controlled ovarian stimulation protocol used, ovarian response obtained). The normality of the variables was determined by the Shapiro-Wilk test (*p*<0.01), and descriptive statistics for non-normal distributions are presented in the form of median and range. The correlation between serum AMH values and the number of oocytes obtained was assessed using Spearman’s correlation coefficient, with a significance level of 5%. ROC (Receiver Operating Characteristic) curves were used to determine AMH cut-off values predictive of poor and exaggerated response.

## RESULTS

The sample’s demographic characteristics and controlled ovarian stimulation cycles features (gonadotrophins doses and number of stimulation days) are summarised in Table 1. Of the 1003 cycles analysed, 453 (45.16%) were IVF cycles, while 550 (54.84%) were ICSI cycles. The Table 2 summarises AMH values and number of oocytes obtained regarding the total sample and each ovarian response subgroup.

**Table 1:** Demographic characteristics and controlled ovarian stimulation cycles features regarding the total sample and each ovarian response subgroup.

	Age (years)	BMI(kg/m^2^)	Gonadotrophin dose (IU)	Duration of stimulation (days)
Total sample (Median [range]) n=1003	35.00 (22.00-40.00)	23.34 (16.59-52.74)	2700.00 (310.00-7200.00)	10.00 (5.00-19.00)
Poor response (Median [range]) (≤3 oocytes obtained) n=200	37.00 (23.00-40.00)	22.50 (17.51-41.91)	3000.00 (1100.00-7200.00)	10.00 (5.00-18.00)
Exaggerated response (Median [range]) (>15 oocytes obtained) n=157	33.00 (23.00-39.00)	23.94 (17.51-41.62)	2250.00 (1000.00-4025.00)	10.00 (7.00-16.00)
Cycle cancelled (Median [range]) (zero oocytes obtained) n=20	35.50 (23.00-40.00)	21.96 (17.51-28.68)	2850.00 (1100.00-4200.00)	9.50 (5.00-14.00)

**Table 2:** AMH values and number of oocytes obtained regarding the total sample and each ovarian response subgroup.

	AMH (ng/mL)	Number of oocytes obtained (n)
Total sample (Median [range]) n=1003	2.01 (0.01-77.24)	8.00 (0.00-48.00)
Poor response (Median [range]) (≤3 oocytes obtained) n=200	0.74 (0.01-17.39)	2.00 (0.00-3.00)
Exaggerated response (Median [range]) (>15 oocytes obtained) n=157	3.92 (0.20-52.52)	19.00 (16.00-48.00)
Cycle cancelled (Median [range]) (zero oocytes obtained) n=20	0.69 (0.01-7.17)	-

The median age of the patients was 35 years, the median AMH value was 2.01 ng/mL and the median number of oocytes obtained was 8.00.

Of the 1003 cycles, 200 resulted in poor ovarian response (19.94%) and 157 in exaggerated ovarian response (15.65%).

In the subgroup of patients who had an excessive ovarian response, the median age (33.0) was lower than those of the other subgroups and those of the whole sample, while the median AMH value (3.92 ng/mL) was higher than those of the other subgroups.

On the other hand, in the subgroup of patients with a poor ovarian response, the median age (37.0) was higher than that of the other subgroups and that of the whole sample, while the median AMH value (0.74 ng/mL) was lower. Women whose cycle was cancelled (zero oocytes obtained) were also assessed separately, and their characteristics are shown in Tables 1 and 2. The median AMH values for this subgroup was 0.69 ng/mL. However, due to the small number of women (n=20), it was not possible to draw any relevant conclusions.

Regarding the type of protocol used, 950 of the controlled ovarian stimulation cycles (94.72%) were carried using a short protocol with a gonadotropin-releasing hormone (GnRh) antagonist and the remaining 53 (5.28%) using a long protocol with a GnRh agonist.

**Figure 1:**
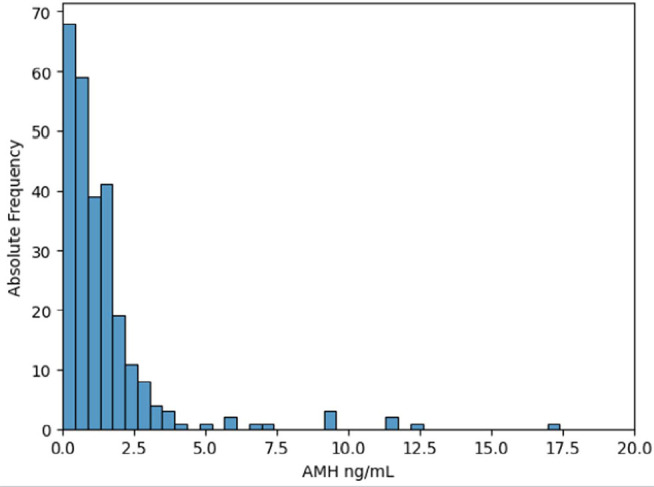
Distribution of AMH values in the subgroup of women with poor ovarian response.

**Figure 2:**
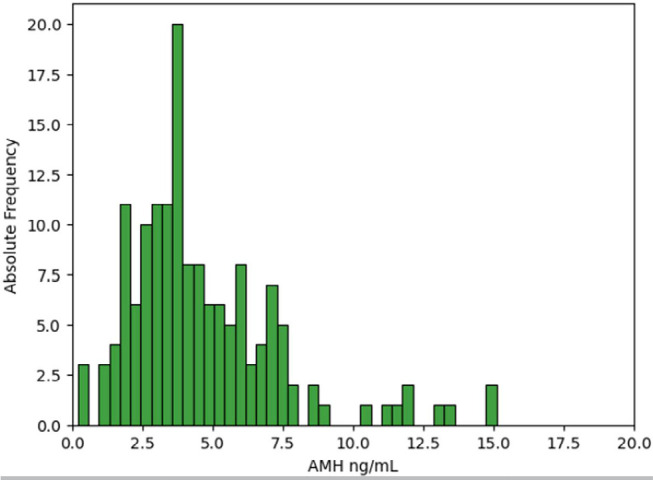
Distribution of AMH values in the subgroup of women with exaggerated ovarian response.

**Figure 3:**
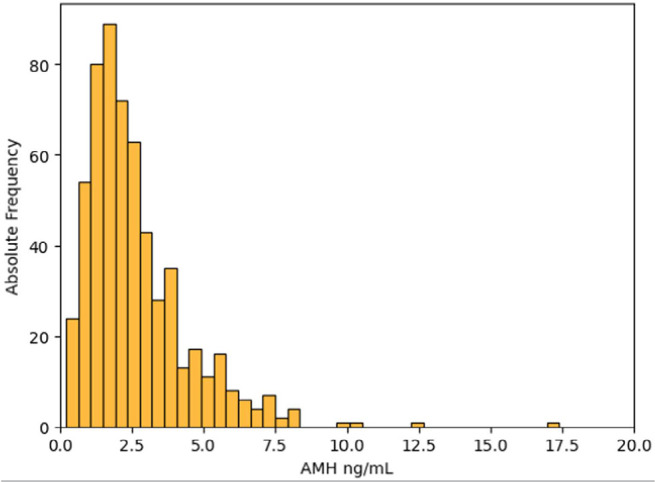
Distribution of AMH values in the subgroup of women with normal ovarian response.

Figures 1, 2 and 3 show the distribution of AMH values according to the ovarian response obtained.

### Correlation between AMH values and the number of oocytes obtained

AMH showed a statistically significant correlation with the number of oocytes obtained. The Spearman’s correlation coefficient (p) calculated was 0.60 (*p*<0.01).

ROC curves were used to calculate the cut-off values, and the predictive values for poor response and exaggerated response were determined separately.

**Figure 4:**
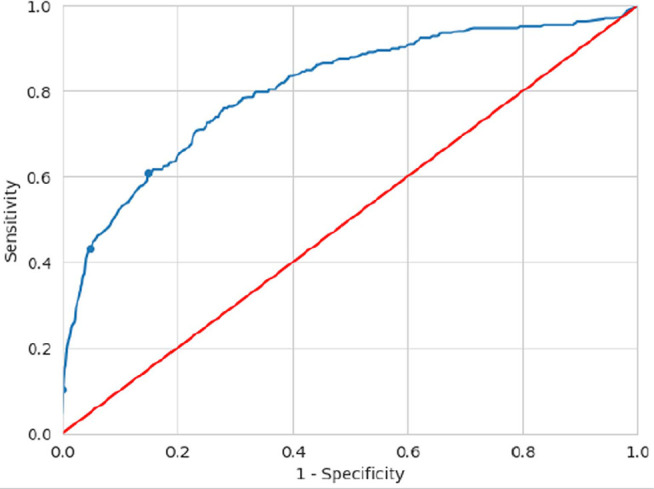
ROC curve differentiating poor ovarian response according to the AMH value [area under the curve (AUC)=0.805; the marked points correspond to the cut-off values shown in Table 3].

The ROC curve for predictive values of poor response is shown in Figure 4. The area under the curve (AUC) is 0.805. Given the parameters of the curve, three different cut-off values were calculated, with different sensitivities and specificities, which are summarised in Table 3.

**Table 3:** AMH cut-off values to differenciate poor ovarian response.

Cut-off values (ng/mL)	Sensitivity (%)	Specificity (%)	Positive predictive value (%)	Negative predictive value (%)
0.18	10.15	100	100	75.56
0.72	43.23	95.13	76.67	82.34
1.23	60.90	85.12	59.80	85.80

The cut-off values presented in the Bologna criteria and POSEIDON criteria were applied to the ROC curve obtained. The POSEIDON criteria (cut-off value 1.2 ng/mL) presented a sensitivity of 57.89%, specificity of 86.06%, with positive and negative predictive values of 59.92% and 85.03%, respectively, regarding the study population. The Bologna criteria (cut-off values 0.5-1.1 ng/mL) presented sensitivity values between 26.32-54.89%, specificity between 88.23-97.97%, with positive and negative predictive values between 62.66-82.35% and 78.70-84.46%, respectively.

**Figure 5:**
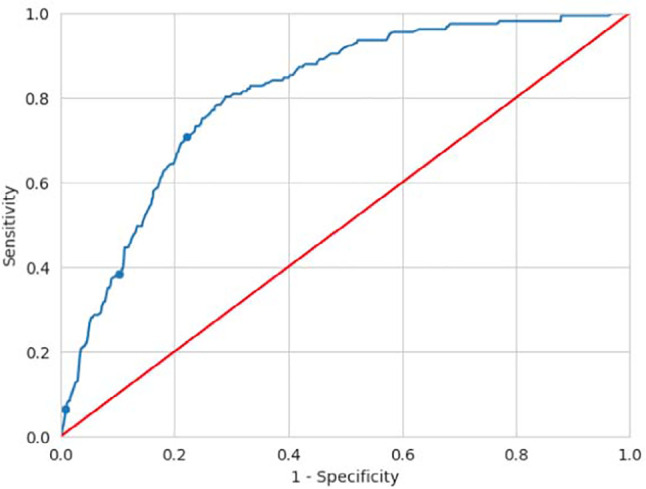
ROC curve differentiating exaggerated ovarian response according to the AMH value [area under the curve (AUC)=0.807; the marked points correspond to the cut-off values shown in Table 4].

The ROC curve for predictive values of exaggerated response is shown in Figure 5. The area under the curve (AUC) is 0.807. Similarly, three different cut-off values were calculated, which are summarised in Table 4.

**Table 4:** AMH cut-off values to differentiate exaggerated ovarian response.

Cut-off values (ng/mL)	Sensitivity (%)	Specificity (%)	Positive predictive value (%)	Negative predictive value (%)
3.09	70.70	77.95	37.25	93.49
4.77	38.22	89.86	41.10	88.71
11.82	6.37	99.17	58.82	85.12

## DISCUSSION

### Correlation between AMH values and number of oocytes obtained

The results show that AMH is positively correlated to the number of oocytes obtained, lower AMH values predicting lower number of oocytes, and higher values predicting a higher number of oocytes. The correlation obtained is in line with those presented by previous studies (La Marca *et al.*, 2010; Akoglu, 2018; Oliveira *et al.*, 2023; Vale-Fernandes *et al.*, 2023a, 2023b).

### Cut-off values

In determining cut-off values, it is important to note that they are expected to be able to demarcate poor responders and over-responders with high precision, without classifying women with the potential for a good ovarian response as poor responders/hyper-responders, since this could lead to the ART technique being abandoned. Therefore, clinicians should be aware that extreme cut-off values are favoured, as they are associated with high specificity (low false-positive rate), even if this implies reduced sensitivity (Ferraretti *et al.*, 2011).

### Poor ovarian response

Analysing Table 3, one can see that the value of 0.18 ng/mL as a cut-off for poor ovarian response offers maximum specificity, however its sensitivity is low, losing its clinical usefulness. On the other hand, the value of 0.72 ng/mL, while maintaining a very high specificity (95.13%), also has a higher sensitivity (43.23%).

For values above 0.72 ng/mL, there is a sharp drop in specificity, which translates into an increase in the number of false positives. Therefore, for the study data, 0.72 ng/mL is the ideal cut-off for differentiating women who will develop a poor ovarian response.

The existent studies on AMH as a predictor for poor ovarian response report cut-off values between 0.099 ng/mL and 1.96 ng/mL. Sensitivity and specificity also vary between 44-97% and 41-100%, respectively (La Marca *et al.*, 2010; Liu *et al.*, 2023). Such differences can be attributed to the following causes:

- The definition of poor response is not consensual in literature, so the number of oocytes that characterise a poor response is variable;
- The sensitivity and specificity required to define a good cut-off point vary from author to author;
- The use of different laboratory kits to measure AMH can also have an impact on the differences observed (Nelson & La Marca, 2011; Nelson, 2013; Iliodromiti *et al.*, 2015).

The variability and lack of standardisation found in the literature reinforces the idea that cut-off values should be specific to each medical centre and its population, since each will have its own definition of poor response and will perform AMH dosages with the kit of its choice.

### Comparison with the cut-offs presented by the POSEIDON criteria and the Bologna criteria

The POSEIDON criteria cut-off value (1.2 ng/mL), when applied to the study population, presented higher sensitivity but compromised specificity.

In the Bologna criteria, a range of cut-off values for low ovarian reserve (<0.5-1.1 ng/mL) is presented, due to the great variability in the literature (Ferraretti & Gianaroli, 2014). Similarly to the POSEIDON criteria, some of the values contained in the range have higher sensitivity but lower specificity than the cut-off calculated in this study; the positive predictive value is also lower and the negative predictive value is very similar. However, it should be noted that the aim of the Bologna criteria was not to distinguish which women are likely to develop poor ovarian response, but rather to reach a consensus on the definition of poor ovarian response in terms of clinical trials (Ferraretti *et al.*, 2011).

That said, the cut-off of 0.72 ng/mL is preferable for the study population compared to those proposed by the published criteria, as it allows differentiation between women who will develop poor ovarian response with greater specificity and higher positive predictive value.

### Exaggerated ovarian response

Analysing Table 4, we can see that the value of 11.82 ng/mL as cut-off for exaggerated ovarian response has maximum specificity. However, once again, it is of limited clinical usefulness as its sensitivity is low. On the other hand, a cut-off of 4.77 ng/mL increases sensitivity without compromising specificity too much. The cut-off of 3.19 ng/mL, despite maximising specificity and sensitivity in combination, has a specificity of 77.95%, which is undesirable as it is a relatively low value. That said, the cut-off of 4.77 ng/mL is preferable for the study population.

To date, few studies have been published on predictive cut-off values for exaggerated ovarian response. The reported values vary between 3.36-4.90 ng/mL, with sensitivities and specificities between 53-91% and 70-95%, respectively (La Marca *et al.*, 2010).

### Study limitations

The main limitation of this study stems from its retrospective design. For this reason, certain characteristics of the sample with a possible influence on the results, such as the dose of exogenous gonadotropins administered or the allocation of patients to the different controlled ovarian stimulation protocols, were not randomised/controlled.

Therefore, future research with a prospective and randomised design could minimise the potential limitations of this study and reduce some of the bias.

## CONCLUSION

The serum AMH value proved to be a good predictor of ovarian response to controlled ovarian stimulation for IVF treatments and is very useful in supporting clinical decision-making. According to the available data and for this population, 0.72 ng/mL was the chosen cut-off value for differentiating poor ovarian response (specificity of 95.13% and sensitivity of 43.23%), and 4.77 ng/mL the chosen cut-off value for differentiating exaggerated ovarian response (specificity of 89.86% and sensitivity of 38.22%). However, these cut-offs should not be used as absolute discriminators, but only to support decision-making. Due to the overlapping of different ovarian responses for the same AMH values, the sensitivity and specificity values obtained for the calculated cut-offs are not excellent and therefore should not be used as sole predictors of response to controlled ovarian stimulation, confirming that AMH alone is not able, for instance, to predict poor ovarian response, unless extremely low values are considered.

The results obtained also highlight the importance of adapting the cut-off values to each medical centre. Medically assisted procreation centers will only be able to provide truly effective advice to their users/patients if they use their own characteristics to predict response/success, allowing a uniform clinical decision, reducing risks, and not excluding any candidate with potential or including them with unrealistic expectations.
